# Supplementary figures and images for: Biophysical modeling and experimental analysis of the dynamics of C. elegans body-wall muscle cells
Source: PLoS Comput Biol. 2025 Jan 27;21(1):e1012318. doi: 10.1371/journal.pcbi.1012318 (PMC11781704; doi:10.1371/journal.pcbi.1012318)

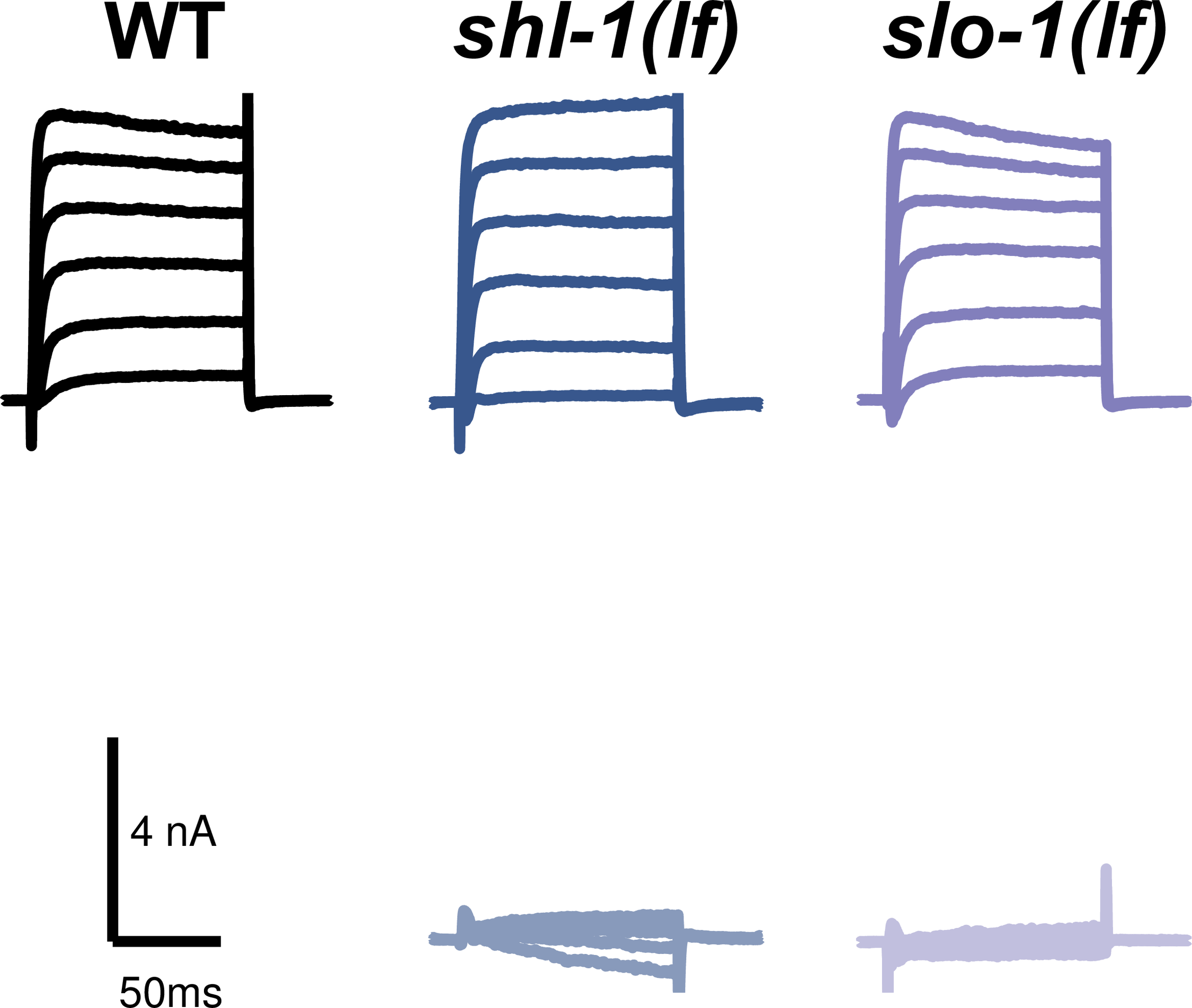

Supplement: S1 Fig — The top section of each column illustrates the currents in different mutants, while the bottom section depicts the ionic currents obtained by subtracting the corresponding mutTIF currents from the total wild type (WT) currents. Only nominal alterations are observed in these two mutants, shl-1(lf) and slo-1(lf). (TIF) [file pcbi.1012318.s001.tif]

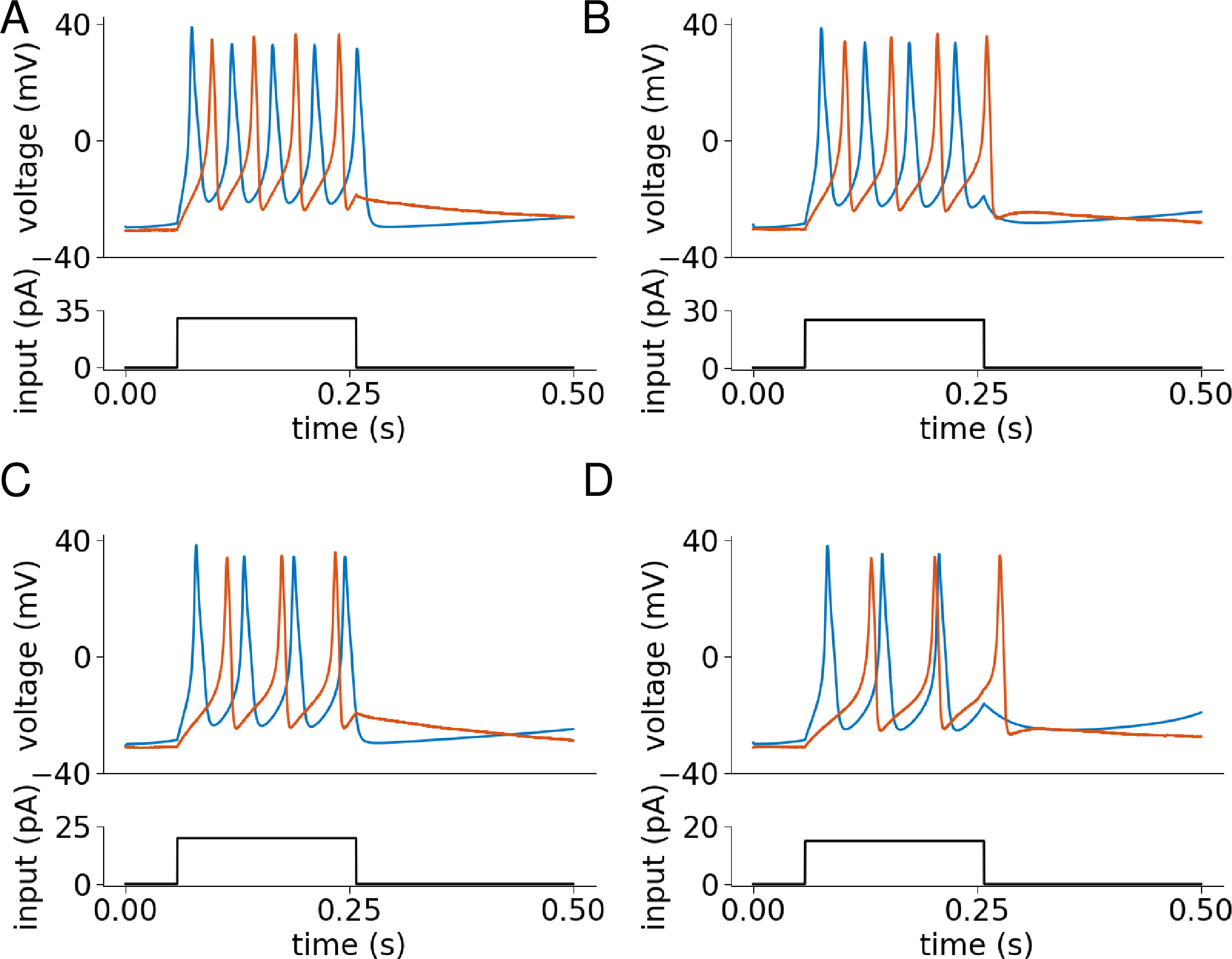

Supplement: S2 Fig — (A-D) These figures present elicited spike trains under varying stimulation currents, ranging from 15 pA to 30 pA in increments of 5 pA. The simulation results are shown in blue, compared to red curves representing experimental data. (TIF) [file pcbi.1012318.s002.tif]

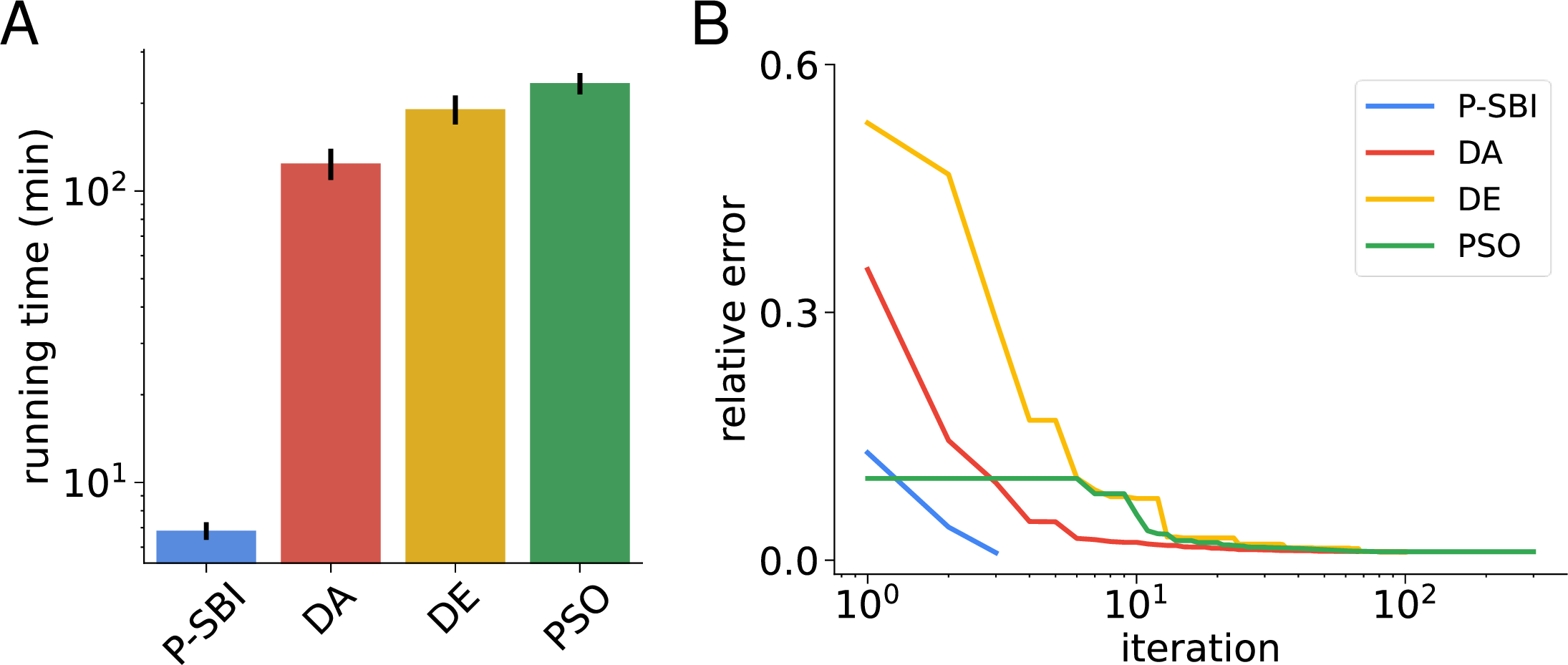

Supplement: S3 Fig — The corner plot displays the marginal and pairwise marginal distributions of the 6-dimensional posterior over gap junction parameters J12 , J23 , … , J56. The true parameter values, marked by red lines, are successfully captured within the high-probability regions of the posterior distribution. (TIF) [file pcbi.1012318.s003.tif]

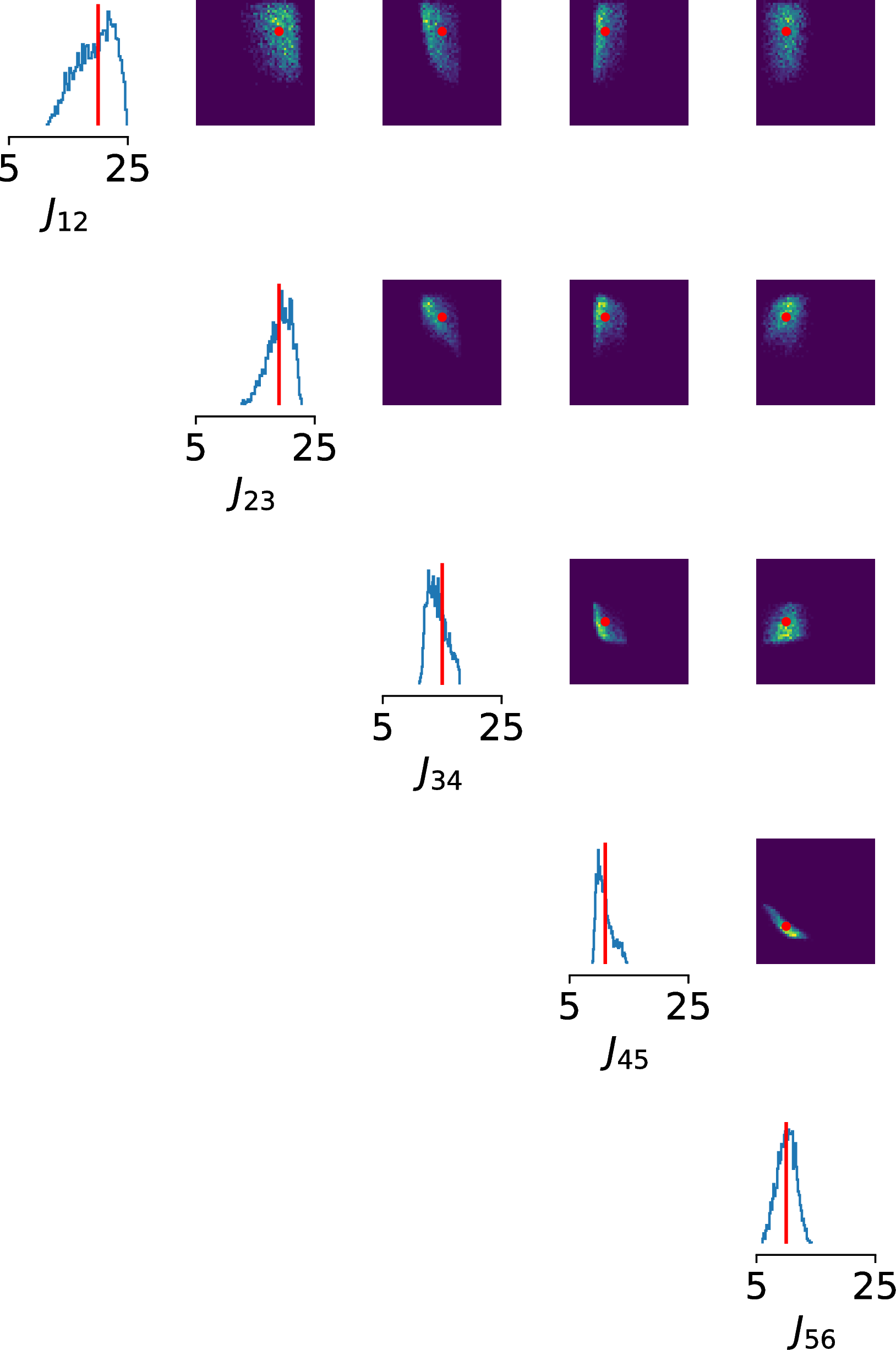

Supplement: S4 Fig — (A) Comparison of running time (in minutes) among four optimization methods for solving the same task in Fig 4E: parallel simulation-based inference (P-SBI), dual annealing (DA), differential evolution (DE), and particle swarm optimization (PSO). The bars represent the average running time across 10 trials, with error bars indicating the standard deviations. (B) Relative error convergence of four optimization methods, plotted as a function of the logarithm of iterations. The relative error is calculated as ||xo-x^o||2||xo||2, where xo is the target solution and x^o is the estimation. The algorithms stop when the relative error falls below 0.01; P-SBI achieves a solution with a relative error less than 0.01 in just 3 iterations. (TIF) [file pcbi.1012318.s004.tif]

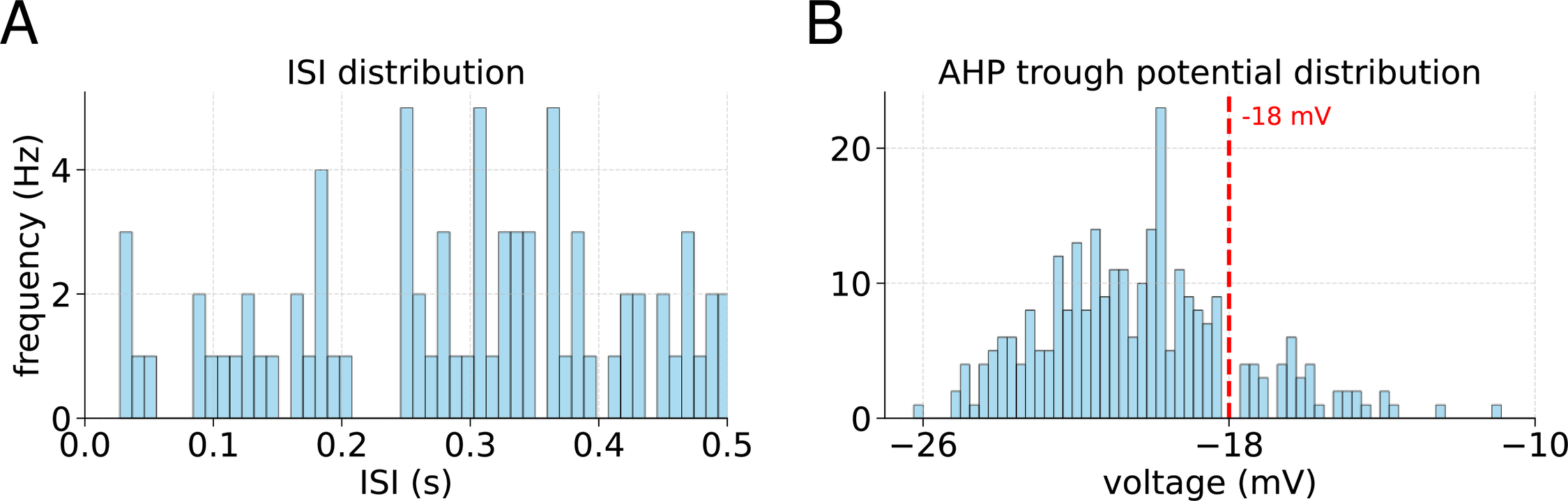

Supplement: S5 Fig — (A) This panel shows the inter-spike interval (ISI) distribution derived from spike trains of multiple body-wall muscle cells. The x-axis represents the ISI in seconds, and the y-axis indicates the number of occurrences for each interval. (B) This panel presents the distribution of afterhyperpolarization (AHP) trough potentials derived from the same spike trains. The x-axis represents the membrane potential (mV), while the y-axis shows the frequency of occurrences within each potential range. (TIF) [file pcbi.1012318.s005.tif]
