# Supplementary material for: Biophysical modeling and experimental analysis of the dynamics of C. elegans body-wall muscle cells
Source: PLoS Comput Biol. 2025 Jan 27;21(1):e1012318. doi: 10.1371/journal.pcbi.1012318 (PMC11781704; doi:10.1371/journal.pcbi.1012318)
Supplement: S1 Appendix — (PDF) [file pcbi.1012318.s006.pdf]

Appendix S1: Equations used in the model simulations.

Voltage-gated Calcium channel: $I_{\text{EGL-19}}$

$$I_{\text{EGL-19}} = g_{\text{EGL-19}} \cdot m^2 \cdot h \cdot (V - E_{Ca})$$
$$\frac{dm}{dt} = \phi_m \frac{m_\infty(V) - m}{\tau_m(V)} \quad \frac{dh}{dt} = \frac{h_\infty(V) - h}{\tau_h(V)}$$
$$m_\infty(V) = \frac{1}{1 + \exp\left(-\frac{V+8-V_{th}}{8.6}\right)} \quad h_\infty(V) = \frac{0.42}{1 + \exp((V + 11 - V_{th})/2)} + 0.28$$
$$\tau_m(V) = 0.4 + \frac{0.7}{\exp(-(V + 5 - V_{th})/15) + \exp((V + 5 - V_{th})/15)} \quad \tau_h = 30$$

Voltage-gated Potassium current: $I_{\text{SHK-1}}$

$$\begin{aligned} I_{\text{SHK-1}} &= g_{\text{SHK-1}} \cdot n^4 \cdot (V - E_K) \\ \frac{dn}{dt} &= \phi_n \frac{n_\infty(V) - n}{\tau_n(V)} \\ n_\infty(V) &= 0.5(\tanh(\frac{V - V_{th} + 15.2}{36.22}) + 1) \\ \tau_n(V) &= 1.18 + \frac{511.78}{1 + \exp((V - V_{th} + 89.3)/21.92)} \end{aligned} \tag{1}$$

Voltage-gated Potassium current: I_{Kr}

$$\begin{aligned} I_{Kr} &= g_{Kr}(1 - q)q_\infty(V)\,(V - E_K) \\ \frac{dq}{dt} &= \frac{q_\infty(V) - q}{\tau_q(V)} \\ q_\infty(V) &= 0.5(\tanh(\frac{V - V_{th} + 32}{5.0}) + 1) \\ \tau_q &= 62 \end{aligned} \tag{2}$$

Calcium dependent potassium current: $I_{\text{SLO-2}}$

$$\begin{aligned} I_{\text{SLO-2}} &= g_{\text{SLO-2}} p^2 z_\infty^3\,(V - E_K) \\ \frac{dp}{dt} &= \phi \frac{p_\infty\left(\left[Ca^{2+}\right]_i\right) - p}{\tau_p\left(\left[Ca^{2+}\right]_i\right)} \\ p_\infty &= \frac{\alpha\left[Ca^{2+}\right]_i^n}{(\alpha\left[Ca^{2+}\right]_i^n + \beta)} \\ \tau_p &= \frac{1}{(\alpha\left[Ca^{2+}\right]_i + \beta)} \\ z_\infty(V) &= \frac{1}{1 + \exp\left(-\frac{V+33.4}{3.2}\right)} \end{aligned} \tag{3}$$

Calcium concentration:

$$\frac{d\left[\text{Ca}^{2+}\right]_i}{dt} = -\frac{I_{Ca}}{2FAd} - \gamma \cdot \left(\left[\text{Ca}^{2+}\right]_i - \left[\text{Ca}^{2+}\right]_r\right)$$
