# Supplementary material for: Biophysical modeling and experimental analysis of the dynamics of C. elegans body-wall muscle cells
Source: PLoS Comput Biol. 2025 Jan 27;21(1):e1012318. doi: 10.1371/journal.pcbi.1012318 (PMC11781704; doi:10.1371/journal.pcbi.1012318)
Supplement: S2 Appendix — (PDF) [file pcbi.1012318.s007.pdf]

S2 Appendix: Network model parameter tuning.

Our algorithm is also applicable to parameter estimation in cellular networks. Previous research has shown gap junctions play a crucial role in coordinating muscle contractions during forward locomotion in *C. elegans*. In particular, muscle cells propagate electrical signals via gap junctions to generate synchronous muscle activity [1–3]. Based on this, we construct a network consisting of six muscle cells sequentially connected by gap junctions that reproduces the synchronization of network activity. In this network, the models of muscle cells are identical, which is described by:

$$C_m \frac{dV_i}{dt} = -I_i^{total}(t) + I_i^{ext} + I_i^{\mathrm{net}}(t). \tag{1}$$

Here, $I_i^{total}(t)$ represents all ion channel currents of the i-th neuron as detailed in Eq. 10; The term I_i^{ext} denotes the external current, the strength of which decreases from the head to the tail muscle cells based on a previous study [2], simulating the gradual reduction of motor neuron signals. Specifically, $I_i^{\mathrm{ext}} = (30 - 5i)$ pA, $i = 1, 2, \cdots, 6$. In addition, $I_i^{\mathrm{net}}(t)$ represents the gap junction current, which is given as follows:

$$I_i^{net}(t) = J_{ij} \sum_{j=1, j\neq i}^{N} (V_j(t) - V_i(t)). \tag{2}$$

Here, J_{ij} represents the connection strength between the i-th and the j-th muscle cells, with $J_{ij} = J_{ji}$. As the muscle cells are connected sequentially, only connections, $J_{12}, J_{23}, \ldots, J_{56}$, are non-zero. In the numerical simulations, we set the connections between muscle cells to be heterogeneous: $J_{12} = 20$ nS, $J_{23} = 19$ nS, $J_{34} = 15$ nS, $J_{45} = 11$ nS and $J_{56} = 10$ nS.

We next use our algorithm estimate the connection parameters based on the simulated network activity. To quantify the network's activity, we first compute the statistical properties of each cell, including firing rates, latency to the first action potentials, the mean and variance of membrane potentials, the mean resting membrane potentials, and mean inter-spike intervals. These statistics are then combined as a vector to define the algorithm's loss function. The simulation results, as shown in S3 Fig, demonstrate that our algorithm performs well in estimating network parameters.

References

1. Wen Q, Po MD, Hulme E, Chen S, Liu X, Kwok SW, et al. Proprioceptive coupling within motor neurons drives *C. elegans* forward locomotion. Neuron. 2012;76(4):750–761.

2. Xu T, Huo J, Shao S, Po M, Kawano T, Lu Y, et al. Descending pathway facilitates undulatory wave propagation in *Caenorhabditis elegans* through gap junctions. Proceedings of the National Academy of Sciences. 2018;115(19):E4493–E4502.

3. Wen Q, Gao S, Zhen M. *Caenorhabditis elegans* excitatory ventral cord motor neurons derive rhythm for body undulation. Philosophical Transactions of the Royal Society B: Biological Sciences. 2018;373(1758):20170370.
