# Supplementary material for: Biophysical modeling and experimental analysis of the dynamics of C. elegans body-wall muscle cells
Source: PLoS Comput Biol. 2025 Jan 27;21(1):e1012318. doi: 10.1371/journal.pcbi.1012318 (PMC11781704; doi:10.1371/journal.pcbi.1012318)
Supplement: S1 Table — (PDF) [file pcbi.1012318.s008.pdf]

S1 table: Default parameters, their corresponding definitions and values.

Parameter	WT	ad1006	n582	sodium-ion-free	Unit	Definition
C_m	22	22	22	22	pF	Membrane capacitance
E_{Ca}	60	60	60	60	mV	Reversal potential for Ca^{2+} channels
E_K	-30	-30	-30	-30	mV	Reversal potential for K^+ channels
E_{Na}	25	25	25	25	mV	Reversal potential for Na^+ channels
E_{Leak}	-16	-16	-16	-16	mV	Reversal potential for Leak current
$g_{\text{EGL-19}}$	19.8	12.8	16.9	19.8	nS	Maximal conductance of Ca^{2+} channels
$g_{\text{SHK-1}}$	37.	37.	37.	37.	nS	Maximal conductance of SHK-1 channels
$g_{\text{SLO-2}}$	3.6	3.6	3.6	8.5	nS	Maximal conductance of SLO-2 channels
g_{Kr}	3.2	7.3	3.5	6.4	nS	Maximal conductance of Kr channels
g_{Na}	0.1	0.1	0.1	0.	nS	Maximal conductance of Na^+ channels
g_{Leak}	0.1	0.1	0.1	0.1	nS	Maximal conductance of Leak channels
ϕ_m	1.2	0.7	0.2	1.2		Constant for adjusting the time scale
γ	0.075	0.075	0.075	0.075	ms^{-1}	Ca^{2+} recovery rate
A	3000	3000	3000	3000	μm^2	Cell surface area
d	0.1	0.1	0.1	0.1	μm	Shell thickness
