# Supplementary material for: Biophysical modeling and experimental analysis of the dynamics of C. elegans body-wall muscle cells
Source: PLoS Comput Biol. 2025 Jan 27;21(1):e1012318. doi: 10.1371/journal.pcbi.1012318 (PMC11781704; doi:10.1371/journal.pcbi.1012318)
Supplement: Attachment — Submitted filename: Response to Reviewers--PCB_final.docx [file pcbi.1012318.s010.docx]

Dear Editor,

We extend our sincere gratitude to you and the reviewers for the invaluable time and constructive feedbacks on our manuscript. We highly appreciate the detailed insights and suggestions about our work. In response, we have meticulously addressed each comment and have made the revisions to our manuscript. Our detailed responses to each comment are provided below, and the changes in the revised manuscript are highlighted in red. We hope that the revised manuscript now satisfies the reviewers’ requirements and hereby resubmit the revised manuscript for publication in PLOS Computational Biology.

Sincerely yours,

Xuexing Du

On behalf of all co-authors

**Responses to the report of Reviewer 1**

*Reviewer #1: This is a solid and well written computational study to model the electrophysiological property of muscle cells of C. elegans, which exhibit a calcium-mediated action potential. The Hodgkin-Huxley-type simulation of voltage gated current and membrane potential dynamics is straightforward and has been used in many prior works. The main development of the current study is to leverage the modern deep learning method and high-speed parallel computing for efficient parameter search. The simulated results appear to agree well with experimental measurements. However, the key methodology implemented in this work is not clearly explained and investigated, making it difficult for readers to understand the limitations and advantages of their computational algorithm.*

**1.** *I do not understand the parallel simulation-based inference algorithm, because the rationale underlying MAP is not explained. The inference neural network was trained on simulated data. However, there is no guarantee that the simulated data would in any way resemble the experimentally measured neural dynamics: the fundamental biophysical ingredients of your model and the prior distribution of the parameters play a critical role here. It is possible that no matter how many simulations you run, the resulting dynamics {*$x_{i}\}$ *remain qualitatively different from the observed data. Related to this point, the convergence of the posterior distribution conditioned on data* $p(\theta|x_{o})$ *is a necessary but not sufficient condition for finding the correct solution space. In fact, under what scenario would this algorithm actually converge?*

**Response:** We thank you for your insightful feedback. We agree with you that the simulated data is not guaranteed to closely match the experimentally observed neural dynamics. We have attempted to resolve this issue by employing two approaches. First, we looked through existing literature related to the ion channel dynamics of *C. elegans* and performed voltage-clamp experiments on both wild-type and various mutants of *C. elegans*, in order to identify and incorporate key ion channels into our model that significantly influence the dynamics of body-wall muscle cells [1-6]. Next, we intentionally set the searched parameter range to be sufficiently broad to account for the physiological variability of ion channel dynamics observed experimentally. Together, these approaches increase the likelihood that the simulated dynamics will qualitatively align with the experimental observations.

As the key part of our algorithm includes the Expectation-Maximization (EM) algorithm, its convergence can be theoretically guaranteed based on the EM algorithm’s convergence, given that the number of simulation trials is sufficiently large [7-8]. In practice, we implemented the following methods to enhance convergence speed and the high accuracy of parameter estimation:

1. **Broad Prior Distribution of Parameters:** We established prior distribution of each parameter that spans the physiological range of ion channel dynamics. This increases the likelihood that the parameter space explored during simulations covers the dynamical range similar to those observed in the experimental data.
2. **Parameter Reduction through Experimental Data:** After identifying the ion channels that significantly impact the dynamics of body-wall muscle cells, we estimate certain parameters for each ion channel separately based on voltage-clamp experimental data. Consequently, we only need to estimate the rest few parameters for each ion channel, thereby facilitating algorithm convergence.
3. **Design of Effective Statistics:** Given the variability of body-wall muscle cells, our goal in fitting experimental data is to capture statistical properties rather than precise trajectory details. Based on this goal, we designed effective statistics and features—such as action potential counts, latency to the first action potential, mean and variance of membrane potentials, mean resting membrane potentials, and mean inter-spike intervals—to reduce the high-dimensional output of voltage trace to a more manageable scale. This strategy reduces problem complexity to facilitate the convergency of algorithm.

To clarify the convergence of the algorithm, we have added the following sentences to our revised manuscript (page 7, lines 210-215):

“…As the key part of our algorithm includes the Expectation-Maximization (EM) algorithm, its convergence can be theoretically guaranteed based on the EM algorithm’s convergence, given that the number of simulation trials is sufficiently large. Specifically, as the total number of simulations $N$ increases, the estimated posterior $q_{\psi}(\boldsymbol{\theta}\mid\mathbf{x}_{o}$) will eventually converge to the posterior distribution conditioned on the observed data $p\left( \boldsymbol{\theta}\mid\mathbf{x}_{o} \right) [49,56]$.”

In addition, we have added the following paragraph to the Discussion section of our revised manuscript (page 19, lines 457-473):

“To improve the performance of our algorithm, we integrate both experimental and numerical approaches. Experimentally, we conduct investigations by reviewing the literature [23, 42, 68, 69] and employing gene manipulation and electrophysiology experiments. Accordingly, we identify the ion channels that influence the dynamics of body-wall muscle cells and select the key channels to incorporate into our model. From the algorithmic standpoint, we implement three strategies to enhance convergence and improve the accuracy of parameter estimation. First, we initialize prior distributions that encompass the full physiological range of ion channel kinetics, enabling the model to explore a sufficiently broad parameter space. Second, we estimate certain parameters for each ion channel separately based on voltage-clamp experimental data. Consequently, we only need to estimate the rest few parameters for each ion channel, thereby facilitating algorithm convergence. Lastly, we develop effective statistics to capture the dynamics we aim to replicate. These statistics—including action potential counts, latency to the first action potential, mean and variance of membrane potentials, mean resting membrane potentials, and mean inter-spike intervals—serve to reduce the complexity of the high-dimensional output while preserving key electrophysiological characteristics.”

**2.** *This work will make a larger impact if the author could discuss whether the current method can be scaled up to simulate not just a single neuron/muscle, but a network of cells. It is also not clear to me when comparing with other methods such as evolutionary algorithms, what is the main advantage of the current method? If speed is a major bottleneck, could you provide a quantitative comparison based on existing literature reports?*

**Response:** We appreciate your thoughtful questions regarding the scalability of the current method and its comparison with other optimization techniques such as evolutionary algorithms. Below, we provide clarification on these points:

1. Scalability to a Network of Cells:

Our algorithm is indeed scalable and well-suited for tuning parameters in cellular networks. Following your suggestion, we have demonstrated the effectiveness of our algorithm in estimating the parameters of a muscle cell network connected via gap junctions. In this network, six muscle cells are sequentially connected. The models of muscle cells are identical, but the connections between the cells are heterogeneous.

In the numerical simulations, we set the parameters of connection strength as the following values: $J_{12}=20 nS$, $J_{23}=19 nS,J_{34}=15 nS, J_{45}=11 nS$ and $J_{56}=10 nS$. In addition, based on a previous experiment [16], each muscle cell receives external input current, the strength of which decreases from the head to the tail muscle cells, simulating diminishing motor neuron signals.

The connection strengths between muscle cells ($J_{12},J_{23},\ldots,J_{56}$) are estimated using our algorithm. The various statistics—including firing rates, membrane potential properties, the mean and variance of membrane potentials, the mean resting membrane potentials, and mean inter-spike intervals—are averaged across the six cells to quantify the network’s output to define the loss function in the algorithm. As shown in Figure R1 (S3 Fig. in our revised manuscript), we have demonstrated that our algorithm also performs well in estimating network parameters.

**Figure R1:** **Posterior distribution.** The corner plot displays the marginal and pairwise marginal distributions of the 6-dimensional posterior over gap junction parameters $J_{12},J_{23},\ldots,J_{56}$. The true parameter values, marked by red lines, are successfully captured within the high-probability regions of the posterior distribution.

We emphasize this in the discussion section (page 19, lines 452-453), where we added the sentence: "Notably, our algorithm can be scaled to estimate parameters not only for individual neurons or muscles but also for networks of cells, as detailed in the S2 Appendix."

The specific experimental details have been included in the newly added content of the manuscript, located in S2 Appendix.

" Our algorithm is also applicable to parameter estimation in cellular networks. Previous research has shown gap junctions play a crucial role in coordinating muscle contractions during forward locomotion in *C. elegans*. In particular, muscle cells propagate electrical signals via gap junctions to generate synchronous muscle activity [1-3]. Based on this, we construct a network consisting of six muscle cells sequentially connected by gap junctions that reproduces the synchronization of network activity. In this network, the models of muscle cells are identical, which is described by:

$$C_{m}\frac{dV_{i}}{dt}=-I_{i}^{\text{total }}\left( t \right)+I_{i}^{\text{ext }}+I_{i}^{\text{net }}\left( t \right).$$

Here, $I_{i}^{\text{total }}(t)$ represents all ion channel currents of the i-th neuron as detailed in Eq. 10; The term $I_{i}^{\text{ext }}$ denotes the external current, the strength of which decreases from the head to the tail muscle cells based on a previous study [2], simulating the gradual reduction of motor neuron signals. Specifically, $I_{i}^{\text{ext }}=\left( 30-5i \right) pA, i=1,2,\cdots,6$. In addition, $I_{i}^{\text{net }}(t)$ represents the gap junction current, which is described as follows:

$$I_{i}^{\text{net }}\left( t \right)=J_{ij}\sum_{j=1,j\neq i}^{N} \left( V_{j}\left( t \right)-V_{i}\left( t \right) \right).$$

Here, $J_{ij}$ represents the connection strength between the i-th and the j-th muscle cells, with $J_{ij}=$ $J_{ji}$. As the muscle cells are connected sequentially, only connections, $J_{12},J_{23},\ldots,J_{56},$ are non-zero. In the numerical simulations, we set the connections between muscle cells to be heterogeneous: $J_{12}=20 nS$, $J_{23}=19 nS, J_{34}=15 nS, J_{45}=11 nS$ and $J_{56}=10 nS$.

We next use our algorithm estimate the connection parameters based on the simulated network activity. To quantify the network's activity, we first compute the statistical properties of each cell, including firing rates, latency to the first action potentials, the mean and variance of membrane potentials, the mean resting membrane potentials, and mean inter-spike intervals. These statistics are then combined as a vector to define the algorithm's loss function. The simulation results, as shown in S3 Fig, demonstrate that our algorithm performs well in estimating network parameters."

2. Efficiency Comparison with Other Algorithms：

Following your suggestion, we have conducted a detailed comparison between our method and other algorithms, including Differential Evolution (DE), Dual Annealing (DA), and Particle Swarm Optimization (PSO) algorithms. We applied these algorithms to our problem and compared their performance to that of our algorithm. As shown in Figure R2 (S4 Fig in our revised manuscript), these algorithms typically converge more slowly, often taking several hours and requiring hundreds of iterations to reach a satisfactory solution, which is consistent with previous studies [9-10]. In contrast, our method demonstrates significantly faster convergence, requiring only 3 iterations to obtain an accurate solution, representing an order-of-magnitude improvement in speed.

**Figure R2:** **Performance comparison of four optimization methods.** (A) Comparison of running time (in minutes) among four optimization methods for solving the same task in Fig.4E: parallel simulation-based inference (P-SBI), dual annealing (DA), differential evolution (DE), and particle swarm optimization (PSO). The bars represent the average running time across 10 trials, with error bars indicating the standard deviations. (B) Relative error convergence of four optimization methods, plotted as a function of the logarithm of iterations. The relative error is calculated as$\frac{\boldsymbol{||x}_{\boldsymbol{o}}\boldsymbol{-}\hat{\boldsymbol{x}}||_{2}}{\left| \left| \boldsymbol{x}_{\boldsymbol{o}} \right| \right|_{2}}$, where $\boldsymbol{x}_{o}$ is the target solution and $\hat{x}$is the estimation. The algorithms stop when the relative error falls below 0.01; parallel SBI achieves a solution with a relative error less than 0.01 in just 3 iterations.

Additionally, our method offers a probabilistic framework for estimating uncertainty in parameter inference, which is especially useful when considering biological variability and experimental noise—common challenges in systems like neuronal and cellular networks. By accounting for the stochastic nature of biological signals, our approach produces more robust and interpretable results.

To emphasize these points, we have added S4 Fig. to our manuscript to display our numerical comparison results. Additionally, we have included the following discussion in our revised manuscript (page 19, lines 492-503).

“…We apply all these algorithms to the parameter tuning of our model and compare their running times, as shown in S4 Fig. Specifically, in the task illustrated in Fig. 4E, we estimate 6-dimensional parameters and set the algorithms to stop when the relative error fell below 0.01. These algorithms converge more slowly, often taking several hours and requiring hundreds of iterations to stop, which is consistent with previous studies [82,83]. In contrast, our method demonstrates significantly faster convergence, requiring only 3 iterations, representing more than an order-of-magnitude improvement in speed. Additionally, our method provides a probabilistic framework for estimating uncertainty in parameter inference. This is beneficial in the presence of biological variability and experimental noise, as demonstrated by the variability observed among body-wall muscle cells in this study.”

**3.** *Related to my major concern. Figure 4E appears to suggest that the posterior is modelled as the multivariate Gaussian distribution. Is this an assumption underlying your model?*

**Response:** Thank you for your comment. While Fig. 4E might give the impression that the posterior is modeled as a multivariate Gaussian distribution, this is not an assumption underlying our model. In our approach, we employ a neural network that learns the posterior probability using a masked autoregressive flow (MAF) method. The MAF is a type of normalizing flow that transforms a simple base distribution (often a multivariate Gaussian) into a complex target distribution through a series of invertible and learnable transformations, which may not necessarily be multivariate Gaussian. To clarify this in the manuscript, we have expanded the original sentence (page 7, pages 195-198): “The neural network learns the posterior probability based on a masked autoregressive flow method [54]” to provide additional details.

The new sentence now reads: “The neural network learns the posterior probability based on a masked autoregressive flow method (MAF) [54]. MAF transforms a simple base distribution, typically a multivariate Gaussian, into a complex target distribution through a series of autoregressive and invertible transformations.”

**4.** *The simulation (Figure 7) predicts burst firing for periodic inputs with a frequency > 4Hz. C. elegans undulation frequency barely exceeds 2Hz for swimming animals, and the worm crawls much slower on agar plate. Thus, this prediction does not appear to be behaviorally relevant. Do the author suggest that motor neurons would fire at a high frequency in order to strongly active the muscle cells? They should make their points clear here.*

**Response:** Thank you for your careful observation regarding the high-frequency action potentials (bursting) predicted in our model. We understand your concern about the behavioral relevance of burst firing, given the undulation frequencies of *C. elegans*. To clarify, while it is true that a single action potential can trigger contraction in nematode muscle cells [2], the undulation frequencies observed in behaviors such as swimming and crawling do not directly correspond to the firing rates of individual muscle action potentials. Instead, these behaviors are driven by the coordinated activity of multiple muscle groups, leading to rhythmic, clustered action potential firing within individual muscle cells. These rhythmic bursts can indeed occur at higher frequencies, ranging from 3.4 Hz to 6.5 Hz [11-12], even though the overall locomotor patterns occur at slower frequencies.

Moreover, it's important to note that *C. elegans* motor neurons, including those like VA5, do not follow classical all-or-none action potential firing. Instead, they generate nonclassical plateau potentials [13], which modulate neurotransmitter release in a graded manner. This graded release subsequently induces classical action potentials in the body wall muscles [2,14].

We have added the following discussion to our revised manuscript to elaborate these points (page 18, lines 432-443): “...These high-frequency bursts are critical for generating the necessary force and coordination for effective movement. While the undulation frequency of *C. elegans* during locomotion is barely exceeds 2 Hz for swimming animals and is even slower during crawling on agar plates, these observed undulation frequencies do not directly result from the firing rate of individual muscle cell. Instead, these behaviors are driven by the coordinated activity of multiple muscle groups, leading to rhythmic, clustered action potential firing within individual muscle cells. These rhythmic bursts can occur at higher frequencies, ranging from 3.4 Hz to 6.5 Hz [65,66], which covers the predicted preferred frequency. Moreover, the burst firing is often driven by plateau potentials in motor neurons, which modulate neurotransmitter release in a graded manner, enabling sustained and synchronized muscle activation during locomotion [40,67].

**Responses to the report of the second Reviewer 2**

*Reviewer #2: This study presents a biophysical model of C. elegans muscle action potentials as well as introducing a new method of parameter fitting for the model. Both are potentially useful for the field. As my expertise lies in C. elegans neuroscience I have focused my comments there. In general, quantitative comparison between measured and simulated action potentials (APs) would help support specific conclusions drawn in the text. In addition, relating the findings particularly about the mutants back to the phenotypes and effects on muscle function in a concrete way would illustrate the power of the student and improves its impact.*

**1.** *Much of the first paragraph does not seem relevant. Creation of human-level intelligent system seems to be a far cry from modeling muscle excitation. Much of the value of the C. elegans stems from analysis of specific genes/constructs mutations in a simple system. A comprehensive biophysical model of muscle activation will be very helpful in this regard. Thus, the focus, in my opinion is more centered on modeling of individual cell activity to study cell elements involved as well a modeling/understanding the simple locomotory systems in C. elegans.*

**Response:** We appreciate your comments and have made the corresponding revisions. Specifically, we have removed the content related to human-level intelligent systems from the manuscript, as it is not directly relevant to the focus of this study. Additionally, we have expanded our discussion on the advantages of analysis and manipulation of specific genes. To ensure clearer logic and coherence, we have rewritten the first paragraph of the Sec. Introduction.

“The nematode *C. elegans* provides a highly accessible model for biological study due to its relatively simple neuronal network, consisting of far fewer neurons and synapses compared to more complex organisms [1]. In addition, due to its genetic simplicity and the ease of performing gene manipulations, this species also serves as an ideal platform for studying specific genes or mutations within a simple system, such as its locomotory circuits [2-4]. Furthermore, the connectome of *C. elegans* has been comprehensively mapped at the cellular level [2,5], providing a valuable and unique resource for both experimental investigations and neuronal network modeling. Despite its simplicity, the *C. elegans* neuronal network possesses remarkable computational efficiency and versatility. This enables the nematode to execute a diverse array of behaviors, including movement, feeding, sleeping, and mating [6-9]. Moreover, this species is capable of adapting its behavior in response to environmental changes and various stimuli, such as hunger, sex, or stress [7,10,11]. These characteristics make *C. elegans* an invaluable model for researching specific neural systems—such as motor, feeding, and olfactory systems—and for understanding the specific cellular elements involved in these functions.”

**2.** *The trace in Fig 1a) is referred to as a graphical representation? This is actual an experimental measurement, correct? That should be explained explicitly. “Representation” is vague. This is simply a baseline measurement of membrane potential in a wild-type animal? Again, please explain briefly in the main text. Do the muscles twitch when they spike? There are no motion artifacts? Or how are they compensated for?*

**Response:** Thank you for your comments and we apologize for the confusion. In fact, the graphical representation is referred to the green worm schematic instead of voltage trace. In addition, the voltage trace in Fig. 1A represents an in vivo recording of spontaneous action potentials from the body wall muscle of a wild-type *C. elegans.* This is actual experimental data showing spontaneous electrical activity under baseline conditions, with no external current stimulation (0 pA during recording).

We have clarified these in the caption of Fig. 1A. “(A) The graphical representation of *C. elegans* body-wall muscle cells, with their electrical activities recorded from experiments in a wild-type animal. The green schematic is an illustration of the *C. elegans*. The red traces represent an in vivo recording of spontaneous action potentials under baseline conditions, with no external current stimulation (0 pA during recording), showcasing both “burst" and “regular" firing patterns. A zoomed-in view of a single spike is shown in the inset, highlighting the distinct contributions of these ion channels.”

Regarding muscle twitching, we did observe minor twitches during the recordings. However, due to the high electrode seal resistance (above GΩ) between glass pipette and cell membrane, there was minimal disruption, and we did not need to compensate for motion artifacts [2]. The stability of the recording ensured that the spontaneous firing we observed is a reliable reflection of muscle electrical activity. We elaborate on this point in the manuscript (page 4, lines 104-111).

“While the primary goal of these recordings was to measure membrane currents and potentials under controlled conditions, we observed some minor muscle twitches during the experiments. However, these movements did not significantly impact the data quality. Due to the high electrode seal resistance (above $G\Omega$) between the glass pipette and the cell membrane, there was minimal disruption to the recordings, and no compensation for motion artifacts was necessary. This level of stability ensured that the spontaneous firing we observed reliably reflected the underlying muscle electrical activity.”

**3.** *Fig1 The two egl-19 lf mutants show different effects on calcium current. Do they have differing phenotypes in terms of effects on muscle function or are they similar? See point in discussion below.*

*Discussion: An advantage of C. elegans is it genetic flexibility to disrupt individual genes etc. in a simple controlled system. As mentioned, the modeling of muscle function presented here could be valuable for assessing effects of specific mutants etc. but there is little attempt to tie the results back to actual muscle function/behavior in a meaningful way. For example, the two egl-19lf mutants show different effects on Ca current that can be understood and explored through the model. How do crawling/muscle function phenotypes compare between these two alleles and how does that relate to what has been demonstrated on a physiological level? A discussion such as this would add weight to the findings and study.*

**Response:** Thank you for your question. To study the role of egl-19 in action potential generation, we used two viable, recessive, partial loss-of-function (lf) alleles, *n582* and ad1006, as *egl-19* null mutants are embryonically lethal. Both *egl-19(n582)* and *egl-19(ad1006)* are mutations in the same gene, leading to dysfunction of the egl-19 channel. Despite some differences in their effects on calcium currents, these alleles exhibit similar phenotypes in terms of muscle function, including flaccid paralysis, slow movement, feeble pharyngeal pumping, and defective egg-laying. These shared phenotypic traits have been previously described in the literature [13]. We have clarified these points in our revised manuscript (page 14, lines 346-354).

“To study the role of *egl-19* in action potential generation, we use two viable, recessive, partial loss-of-function (lf) alleles, *n582* and *ad1006*, as *egl-19* null mutants are embryonically lethal. Both *egl-19(n582)* and *egl-19(ad1006)* are mutations in the same gene, leading to dysfunction of the *egl-19* channel. Despite some differences in their effects on calcium currents, these alleles exhibit similar phenotypes in terms of muscle function, including flaccid paralysis, slow movement, feeble pharyngeal pumping, and defective egg-laying. These shared phenotypic traits have been previously described in the literature [42]. We then use our model to explore the impact of these mutations on action potentials in body-wall muscle cells.”

**4.** *Pg 9: “We note that when the model contains only the four previously mentioned ion channels, the action potentials exhibit premature repolarization compared to experimental data.” Is that shown some place? Would be good to illustrate the difference for the reader can fully understand.*

**Response:** Following your suggestion, we have now included several figures illustrating the differences between the premature repolarization observed in the model and the experimental data. These figures have been added to the supplementary materials for clarity, as shown in Figure R3 (S2 Fig in our revised manuscript). Additionally, we have updated the manuscript to reference these figures (page 11, lines 299-301): “We note that when the model contains only the four previously mentioned ion channels, the action potentials exhibit premature repolarization compared to experimental data, as shown in S2 Fig.”

**Figure R3:** **Elicited spike trains across varying stimulation currents with four primary ion channels.** (A-D) These figures present elicited spike trains under varying stimulation currents, ranging from 15 pA to 30 pA in increments of 5 pA. The simulation results are shown in blue, compared to red curves representing experimental data.

**5.** *pg 14 “Specifically, under a 30 pA current injection, the average action potential amplitude in wild-type cells is 61.6 mV, as shown by the red curve in Fig. 4A. In contrast, in the egl-19(ad1006,lf) mutants, the amplitude is less than half of this value, as depicted by the red curve in Fig. 5A.”This is an observation of individual experimental trials. Measurement of numbers action potential and comparison of mean would be more convincing.*

**Response:** Thank you for your suggestion. In response, we performed statistical calculations and used bar plots to compare action potential amplitudes between wild-type and *egl-19(ad1006, lf)* mutants across multiple individuals, as shown in Figure R4 (Fig. 5(B-D) in our revised manuscript). Our calculations show that the amplitude in WT cells is 59.61 ± 0.35 mV, while in ad1006 mutants it is 29.98 ± 0.92 mV, approximately half that of wild-type cells. In addition, we also compared the inter-spike intervals and mean resting potentials between the two groups.

To clarify these, we have added the following to our revised manuscript (page 14, lines 359-362): “…Specifically, under a 30 pA current injection, the average action potential amplitude in *egl-19(ad1006,lf)* mutants is approximately half that of wild-type cells, while the average inter-spike interval is significantly prolonged, as illustrated in the bar graph in Fig. 5(B-C).”

**Figure R4: Statistical comparison of action potential measurements between wild-type (WT) and egl-19(ad1006,lf) mutants.** The average amplitude and inter-spike intervals in *egl-19(ad1006,lf)* mutants are significantly different than those in WT. Amplitude (mean$\pm$ SEM):$WT\left( \exp\right),59.61\pm0.35mV;WT\left( \mathrm{sim} \right),59.19\pm2.31mV;$ *ad1006*(exp), $29.98\pm0.92mV$; *ad1006* (sim), $32.81\pm0.04 mV$. Inter-spike interval: $WT\left( \exp\right)$, $53.28\pm6.39\text{ }ms;WT\left( \mathrm{sim} \right)$, $48.81\pm1.29\text{ }\text{m}s;ad1006\left( \exp\right),168.51\pm11.22\text{ }\text{m}s;ad1006\left( \mathrm{sim} \right),116.58\pm0.12\text{ }\text{m}s$. The number of animals recorded per genotype: $WT:n=6$; *ad1006* (exp): $n=4$.

**6.** *Pg 14“Notably, the mutant model agrees with the shape of action potentials under constant current injections, as shown in Fig. 5B.”I do not see where the model is compared to the actual data. Overlaying the model trace on top of a number of measured action potentials (or the means of a number of action potentials) would be more convincing. Likewise of measurement of AP frequency comparing measured vs simulated would be more convincing.*

**Response:** Thank you for your valuable suggestion regarding the comparison between the mutant model and experimental data. In response, we have updated Fig. 5 by overlaying the simulated action potentials onto the measured traces from four *egl-19(ad1006,lf)* mutants, as shown in Figure R5 (Fig. 5A in our revised manuscript). From Figure R5, we observed that the voltage trace of four mutants has a large variability and our simulated voltage trace falls within the experimental range.

This is further verified quantitively by statistical comparisons of action potential amplitudes and inter-spike intervals between the wild-type and mutant models in Fig. 5(B-C). We have also revised the relevant section of the manuscript (page 14, lines 359-364). “…Specifically, under a 30 pA current injection, the average action potential amplitude in *egl-19(ad1006,lf)* mutants is approximately half that of wild-type cells, while the average inter-spike intervals is significantly prolonged, as illustrated in the bar graph in Fig. 5(B-C). Notably, the mutant model agrees with the shape of action potentials under constant current injections, as shown in Fig. 5(A-C).”

**Figure R5:** (A) Left panel: Experimental (grey) and simulated (blue) voltage responses of *C. elegans* muscle cells from *egl-19(ad1006,lf)* mutants under a constant input current of 30 pA. Experimental traces from four mutants are shown, demonstrating significant variability among individuals. Right panel: Overlay of individual action potentials from different mutants (grey), aligned with simulated spikes (blue), extracted from the traces on the left. (B-C) The average amplitude and inter-spike intervals in *egl-19(ad1006,lf)* mutants are significantly different than those in WT. Amplitude (mean$\pm$ SEM):$WT\left( \exp\right),59.61\pm0.35mV;WT\left( \mathrm{sim} \right),59.19\pm2.31mV;$ *ad1006*(exp), $29.98\pm0.92mV$; *ad1006* (sim), $32.81\pm0.04 mV$. Inter-spike interval: $WT\left( \exp\right)$, $53.28\pm6.39\text{ }ms;WT\left( \mathrm{sim} \right)$, $48.81\pm1.29\text{ }\text{m}s;ad1006\left( \exp\right),168.51\pm11.22\text{ }\text{m}s;ad1006\left( \mathrm{sim} \right),116.58\pm0.12\text{ }\text{m}s$. The number of animals recorded per genotype: $WT:n=6$; *ad1006* (exp): $n=4$.

**7.** *Comparing Fig 5C and D simulations of action potentials in elg-19(n582,lf) do not match the shape of measured action potentials in experiments. Again overlaying the simulated AP with a number of experimental measured APs would add in this comparison. This is not commented on at all and should be addressed. Why doesn’t it match the AP profile? what else might be going on here? The simulation does appear to predict the slower firing rate but again there is no quantitative comparison of measured firing rate vs simulated firing rate.*

**Response:** Thank you for your valuable suggestion. In response, we have overlaid the simulated and experimentally measured action potentials, as shown in Figure R6 (Fig. 5(D-E) in our revised manuscript). While discrepancies exist between the shapes of the overlaid experimental and numerical results, our primary objective is not to achieve a perfect shape fit but to focus on changes in the firing rate. Based on experimental measurements of calcium ion currents in mutants, we adjusted only the time constant $\tau_{m}$ in our model and found that this adjustment sufficiently captures the changes in firing rate. Depicting the precise shape of the action potential may require considering other channel dynamics. Further analysis of additional experimental data revealed that in most *egl-19(n582,lf)* mutants, the firing rate decreases to zero. By further increasing $\tau_{m}$ in the model, we can more accurately align the trajectories. Additionally, we revised the relevant section of the manuscript to explain this observation (page 14, lines 367-376).

“Namely, experimental data indicate an increase in the activation time constants of the EGL-19 channel, denoted as $\tau_{m}$ in these mutant models, as shown in Fig. 1C. Our simulations, depicted in Fig. $5$(D-E), replicate this observation by increasing the $\tau_{m}$ values several-fold. As shown in Fig $5$F, with each incremental increase in $\tau_{m}$, it approaches $\tau_{n}$. When $\tau_{m}$ is increased 20 -fold, it surpasses $\tau_{n}$ throughout most of the firing voltage range. This adjustment significantly reduces the firing rate of the cells compared to wild-type, even leading to a marked inability to generate action potentials, as illustrated in Fig. $5$(D-E)$.$ Similarly, under the same current injection conditions in experiments, we observe that *egl-19(n582,lf)* mutants rarely fire, with only a few neurons sparsely spiking, as shown in Fig. $5$(D-E).”

**Figure R6:** Left panel: Comparison of experimental voltage responses from *egl-19(n582,lf)* mutants and simulated data with an altered time constant $\tau_{m}$ (5.5-fold). Right panel: Simulated voltage responses with an altered time constant $\tau_{m}$ (20-fold) compared to *egl-19(n582,lf)* mutants that did not exhibit action potentials. Data from four mutants are displayed in grey, with the simulated trace shown in green.

**8.** *Fig 6 Again comparison of AP via with overlayed individual AP traces and quantitative measurement of AP frequency would be helpful.*

**Response:** Thank you for your suggestion to include overlaid individual action potential (AP) traces and a quantitative comparison of AP frequency. In response, we have incorporated these comparisons in Figure R7 **(**Fig. 6(A-C) in our revised manuscript**)**. We now provide an overlay of individual experimental AP traces from wild-type (WT) cells (in gray) with the simulated APs (in blue). From Figure R7, we observed that the voltage trace of experimental data in a sodium-ion-free solution has a large variability and our simulated voltage trace falls within the experimental range. This is further verified quantitively by statistical comparisons of action potential amplitudes and inter-spike intervals between the experimental and simulated data.

**Figure R7:** **Investigating the impact of potassium channel SLO-2 on neuronal dynamics in a sodium-ion-free environment.** (A) Left panel: Voltage traces recorded experimentally (gray) from WT in response to a 30 pA input current applied for 2 seconds, alongside corresponding simulated traces (blue) in a sodium-ion-free solution. Right panel: Overlay of individual action potentials (grey), aligned with simulated spikes (blue), extracted from the traces on left. (B-C) Statistical comparison between experimental (exp) and simulated (sim) data. Amplitude: exp, $45.66\pm1.37 mV$; sim, $54.55\pm0.79 mV$. Inter-spike-interval: exp, $129.33\pm10.30 ms$; sim, $154.17\pm11.01 ms$. The number of animals recorded:$n=4$. Error bars represent the standard error of the mean (SEM).

We have also revised the relevant section of the manuscript (page 16, lines 378-391).

“When substituting extracellular sodium ions $Na^{+}$ with N-methyl-D-glucamine (NMDG), notable changes occur in the inter-spike-intervals of muscle cells. In our quest to pinpoint the channels responsible for these observations, we scrutinize the voltage clamp data pertaining to all ion channels involved in action potential generation. The removal of extracellular sodium ions first leads to a cessation of the NCA sodium leak current. Our investigation further reveals that the SLO-2 channel exhibits an increased steady-state value in the absence of $Na^{+}$ compared to the normal condition. The *slo-2* encodes a subunit of the $K^{+}$ channel that is modulated by calcium and chloride ion concentrations [39]. The absence of extracellular $Na^{+}$ induces a shift in the dynamics of the SLO-2 channel, contributing to the altered action potentials. After calibrating the SLO-2 and NCA channel parameters in our model by modifying the maximum conductance of SLO-2 and setting the NCA current to zero, we find that the simulation results closely align with the experimental data, as shown in Fig. 6A. A statistical comparison between the simulation and experimental results is provided in Fig. 6(B-C).”

**9.** *Pg 17 How is the ISI threshold of burst set to 200ms? Is there a clear bimodal distribution in ISI values that clearly delineate burst from normal firing or is it a continual distribution with an arbitrary threshold? This needs to be explained more clearly and justified.*

**Response:** Thank you for your valuable comment. The threshold of 200 ms for defining burst activity was chosen based on the analysis of the ISI distribution, as shown in Figure R8 (S5 Fig. in our revised manuscript). While the distribution is not perfectly bimodal, there is a noticeable gap in the distribution. The 200 ms threshold was set based on this separation, providing a reasonable cutoff to differentiate between burst and non-burst firing.

Additionally, we have added a detailed explanation in our revised manuscript (page 16, 413-419).

“Notably, the body-wall muscle cells of *C. elegans* exhibit a distinct preferred frequency at around 4.7 Hz. To compare with experimental data, we perform a statistical analysis of ISIs across 10 spike trains recorded from three wild-type *C. elegans* individuals, as shown in S5 Fig. While the distribution is not perfectly bimodal, there is a noticeable gap in the distribution. We set a 200 ms threshold based on this separation, providing a reasonable cutoff to differentiate between burst and regular firing patterns.”

**Figure R8:** **Spike train analysis of body-wall muscle cells: ISI distribution.**

The figure shows the inter-spike interval (ISI) distribution derived from spike trains of multiple body-wall muscle cells. The x-axis represents the ISI in seconds, while the y-axis indicates the count of occurrences for each interval.

**10.** *Pg 17 The measured burst frequency of 4.8 Hz is surprisingly close to the burst cutoff time of 200 ms (i.e., 5Hz, although I also don’t fully understand how it could be lower than 5Hz?). Does the burst frequency depend on the ISI burst threshold time? It seems like it might or needs to be shown otherwise. If so, a very strong justification of the threshold time is needed. Without further explanation of the burst frequency measurement, claims about the optimal response frequency of of muscles cells cannot be justified.*

**Response:** We appreciate your insightful observation regarding the burst frequency and its relationship to the ISI threshold. As we discussed in Response 9, the choice of the cutoff time is based on the analysis of the ISI distribution, where there is a clear gap around 200 ms. However, the cutoff threshold does not necessarily determine the burst frequency, but it provides a lower bound of burst frequency: if there are multiple spikes within 200 ms, the calculated frequency may exceed 5 Hz rather than being around 5 Hz. But we agree with you that the measured burst frequency is dependent on the ISI threshold, which defines burst activity pattern.

In addition, we apologize that the detailed calculation method of burst frequency was not fully explained in the original manuscript, which led to the confusion regarding the measured burst frequency being lower than 5 Hz. In addition to the ISI threshold, we incorporated an extra criterion to define burst activity based on the reference [17]: a burst event is accompanied by a sustained depolarization phase (platform phase) with a voltage above −18 mV, as shown in Figure R9. The choice of −18 mV was based on our analysis of the afterhyperpolarization (AHP) trough potential distribution of each spike, as shown in Figure R10 (S5 Fig in our revised manuscript). Therefore, in a burst event, the burst duration was defined as the period between the membrane potential rising above −18 mV before the first spike and decreasing back to −18 mV after the last spike. In cases where the membrane potential consistently remains above −18 mV, the burst duration was determined by the inflection point prior to the first spike as the onset and the inflection point following the last spike as the offset. In this way, we have obtained the frequency of some burst events slightly lower than 5 Hz.

**Figure R9:** **A representative spontaneous action potential curve in WT cells, showing both “burst” and“regular” firing modes. The firing rates of cells in the“burst” firing mode are calculated.**

**Figure R10:** **Spike train analysis of body-wall muscle cells: AHP trough potential distribution.** This figure presents the distribution of afterhyperpolarization (AHP) trough potentials derived from spike trains of multiple body-wall muscle cells. The x-axis represents the membrane potential (mV), while the y-axis shows the frequency of occurrences for each potential range.

In the revised manuscript, we have included a more detailed explanation of this method to ensure a clearer understanding of how the burst frequency is calculated (Page 17, lines 420-431).

“Additionally, we apply an extra criterion to define burst activity based on the reference [64]: a burst event is accompanied by a sustained depolarization phase (platform phase) with a voltage above -18 mV, as shown in Fig. 7C. The choice of −18 mV is based on our analysis of the afterhyperpolarization (AHP) trough potential distribution of each spike, as shown in S5 Fig. Therefore, in a burst event, the burst duration is defined as the period between the membrane potential rising above −18 mV before the first spike and decreasing back to −18 mV after the last spike. In cases where the membrane potential consistently remains above −18 mV, the burst duration is determined by the inflection point prior to the first spike as the onset and the inflection point following the last spike as the offset. This dual criterion—combining ISI and depolarization plateau—ensures a precise identification of burst events, distinguishing them from random spike occurrences.”

[1]. Liu, Ping, et al. "Genetic dissection of ion currents underlying all-or-none action potentials in *C. elegans* body-wall muscle cells." The Journal of physiology 589.1 (2011): 101-117.
[2]. Gao, Shangbang, and Mei Zhen. "Action potentials drive body wall muscle contractions in Caenorhabditis elegans." Proceedings of the National Academy of Sciences 108.6 (2011): 2557-2562
[3]. Nicoletti, Martina, et al. "Biophysical modeling of C. elegans neurons: Single ion currents and whole-cell dynamics of AWCon and RMD." PloS one 14.7 (2019): e0218738.
[4]. Liu, Qiang, et al. "C. elegans AWA olfactory neurons fire calcium-mediated all-or-none action potentials." Cell 175.1 (2018): 57-70.
[5]. Jospin, Maëlle, et al. "The L-type voltage-dependent $Ca^{2+}$ channel EGL-19 controls body wall muscle function in Caenorhabditis elegans." The Journal of cell biology 159.2 (2002): 337-348.
[6]. Yan, Zhenzhen, et al. "Caenorhabditis elegans body wall muscles sense mechanical signals with an amiloride-sensitive cation channel." Biochemical and Biophysical Research Communications 527.2 (2020): 581-587.
[7]. Deistler, Michael, Pedro J. Goncalves, and Jakob H. Macke. "Truncated proposals for scalable and hassle-free simulation-based inference." Advances in Neural Information Processing Systems 35 (2022): 23135-23149.
[8]. Moon, Todd K. "The expectation-maximization algorithm." IEEE Signal processing magazine 13.6 (1996): 47-60.
[9]. Buhry, Laure, Michele Pace, and Sylvain Saïghi. "Global parameter estimation of an Hodgkin–Huxley formalism using membrane voltage recordings: Application to neuro-mimetic analog integrated circuits." Neurocomputing 81 (2012): 75-85.
[10]. Buhry, Laure, et al. "Automated parameter estimation of the Hodgkin-Huxley model using the differential evolution algorithm: application to neuromimetic analog integrated circuits." Neural computation 23.10 (2011): 2599-2625.
[11]. Liu, Ping, Bojun Chen, and Zhao-Wen Wang. "Postsynaptic current bursts instruct action potential firing at a graded synapse." Nature communications 4.1 (2013): 1911.
[12]. Gao, Shangbang, et al. "Excitatory motor neurons are local oscillators for backward locomotion." Elife 7 (2018): e29915.
[13]. Liu, Ping, Bojun Chen, and Zhao-Wen Wang. "SLO-2 potassium channel is an important regulator of neurotransmitter release in Caenorhabditis elegans." Nature communications 5.1 (2014): 5155.
[14]. Liu, Qiang, Gunther Hollopeter, and Erik M. Jorgensen. "Graded synaptic transmission at the Caenorhabditis elegans neuromuscular junction." Proceedings of the National Academy of Sciences 106.26 (2009): 10823-10828.
[15]. Lee, Raymond YN, et al. "Mutations in the α1 subunit of an L‐type voltage‐activated $Ca^{2+}$ channel cause myotonia in Caenorhabditis elegans." The EMBO journal (1997).
[16]. Xu, Tianqi, et al. "Descending pathway facilitates undulatory wave propagation in Caenorhabditis elegans through gap junctions." *Proceedings of the National Academy of Sciences* 115.19 (2018): E4493-E4502.
[17]. Beurrier, Corinne, et al. "Subthalamic nucleus neurons switch from single-spike activity to burst-firing mode." Journal of Neuroscience 19.2 (1999): 599-609.
